# Efficacy and toxicity of whole brain radiotherapy in patients with multiple cerebral metastases from malignant melanoma

**DOI:** 10.1186/1748-717X-7-130

**Published:** 2012-08-02

**Authors:** Henrik Hauswald, Jan-Oliver Dittmar, Daniel Habermehl, Stefan Rieken, Florian Sterzing, Jürgen Debus, Stephanie E Combs

**Affiliations:** 1 Department of Radiation Oncology, University of Heidelberg, INF 400, Heidelberg, 69120, Germany

**Keywords:** Malignant melanoma, Brain metastases, Irradiation, Radiotherapy, WBRT, Whole brain radiotherapy

## Abstract

**Background:**

To retrospectively access outcome and toxicity of whole brain radiotherapy (WBRT) in patients with multiple brain metastases (BM) from malignant melanoma (MM).

**Patients and methods:**

Results of 87 patients (median age 58 years; 35 female, 52 male) treated by WBRT for BM of MM between 2000 and 2011 were reviewed. Total dose applied was either 30 Gy in 10 fractions (n = 56) or 40 Gy in 20 fractions (n = 31). All but 9 patients suffered from extra-cerebral metastases. Prior surgical resection of BM was performed in 18 patients, salvage stereotactic radiosurgery in 13 patients.

**Results:**

Mean follow-up was 8 months (range, 0–57 months), the 6- and 12-months overall-(OS) survival rates were 29.2% and 16.5%, respectively. The median OS was 3.5 months. In cerebral follow-up imaging 6 (11) patients showed a complete (partial) remission, while 11 (17) patients had stable disease (intra-cerebral tumor progression). In comparison of total dose, the group treated with 40 Gy in 20 fractions achieved a significant longer OS (p = 0.003, median 3.1 vs. 5.6 months). Furthermore, DS-GPA score (p < 0.001) as well as RPA class (p < 0.001) influenced significantly on OS and patients had a significantly longer OS after surgical resection (p = 0.001, median 3.0 vs. 5.8 months, multivariate p = 0.007). Having extra-cerebral metastases didn't significantly impact on OS (p = 0.21).

**Conclusion:**

Treatment of BM from MM with WBRT is tolerated well and some remissions of BM could be achieved. An advantage for higher treatment total doses was seen. However, outcome is non-satisfying, and further improvements in treatment of BM from MM are warranted.

## Introduction

The malignant melanoma accounts for approximately 4.3% of all cancers in Germany in females and 3.2% in males, respectively and causes around 1% of all cancer deaths. In the last 30 years, the age standardized incidence rates in Germany tripled [1]. Furthermore, the risk of development of brain metastases is stage dependent and as high as 46% in stage IV [2-6]. The median survival is short and reported to be about 4–5 months [7,8]. In case of brain metastases surgical resection should be evaluated, even though brain metastases might be too small for resection or simply too frequent [9]. The standard of care in brain metastases from malignant melanoma includes WBRT delivered as 30 Gy in 10 fractions, or 40 Gy in 20 fractions [10-17]. This results in a median survival of 3–5 months [7,10-17]. Due to the limited efficacy of WBRT, and due to the advent of high precision radiosurgery techniques, radiosurgery (SRS) has been established for subgroups of patients with 1–3 brain metastases. It has been shown that in these patients WBRT plus surgery or SRS leads to an increase of loco-regional control compared to local treatments alone, however, overall survival is unaltered [18]. However, in this analysis, several tumor types with high numbers of breast or lung tumors were included. Several other groups have reported high efficacy of SRS for patients with malignant melanoma brain metastases: Seung et al. treated 140 lesions in 46 patients, with progression-free rates of 86% at 6 and 76% at 12 months, respectively [19,20]. Grob and co-workers published a local control rate of 98% at 3 months for 56 metastases in 35 patients treated with SRS only, without additional WBRT. Median survival was longer in patients with single lesions, i.e. 7.5 months versus 4 months [21]. Our own data published previously was based on 64 patients treated for 122 lesions; in this group, local control was 81% at 12 months, and median survival after treatment was 10.6 months [22]. Another approach would be stereotactic brachytherapy using 125-iodine seeds in solitary brain metastases, giving the advantage of histological evaluation [23]. Published prognostic factors include RTOG-RPA class as well as Karnofsky Performance score (KPI), Disease-Specific Graded Prognostic Assessment Score (DS-GPA-score), number of brain metastases and pretreatment lactase-dehydrogenate level [11,24-27]. However, in some cases, SRS is not possible, or multiple metastases (>3) are present, and WBRT is indicated. In the present work we focused on patients with cerebral metastases from malignant melanoma to evaluate treatment outcome and toxicity.

### Patients and methods

#### Patient characteristics

Between 2000 and 2011 in total 87 patients (median age 58 years; 35 female, 52 male) with multiple brain metastases from malignant melanoma were treated at the Department of Radiation Oncology at the University Hospital of Heidelberg. Prior surgical resection of brain metastases was performed in 18 patients and salvage SRS in 13 patients during the course of their disease. Nine patients had metastases to the brain only, all other additionally multiple extra-cerebral metastases. A summary is found in Table 1.

**Table 1:** Patient characteristics

**Patient characteristic**	**No. of patients**	**Percentage**
**Gender**		
Male	52	60
Female	35	40
**Clark level**		
II	1	1
III	14	16
IV	31	36
V	3	3
n. a.	38	44
**Distant metastases**		
Extra-cerebral	75	86
Only cerebral	9	10
n. a.	3	3
**RPA class**		
1	4	5
2	55	63
3	13	15
n. a.	15	17
**DS-GPA score**		
0	12	14
1	40	46
2	18	21
3	1	1
4	1	1

#### Radiotherapy and follow-up

Radiotherapy was performed as a WBRT delivered in total doses of either 30 Gy in 10 fractions (n = 56; 3 Gy / fraction, 5 fractions a week) or 40 Gy in 20 fractions (n = 31; 2 Gy / fraction, 5 fractions a week) and administered by opposing lateral 6-MeV photon beams. An additional boost irradiation was performed in 8 patients (SRS n = 2, 3D-CRT n = 6). All patients were followed primarily by clinical examinations (n = 41), or by additional imaging procedures as CT or MRI (n = 46). Of these 46 patients with follow-up imaging procedure 24% (n = 11) had surgery prior to WBRT and 11% (n = 5) a boost irradiation. Furthermore, salvage SRS was carried out in 24% of these patients (n = 11).

#### Evaluation and statistics

Statistical analyses were carried out with SPSS statistical package (SPSS Inc., Chicago, IL, U.S.A.) using log-rank test (Mantel-Cox), Kaplan-Meier’s estimation for overall survival (OS), Chi-square test (Pearson and Fisher exact test) and multivariate Cox-regression analysis (backwards stepwise, p out >0.1, factors included: total dose of irradiation (30 vs. 40 Gy), prior surgical resection, age (>/= or <58 years), GPA-score and extra-cerebral metastases). Significance was defined as p-value <0.05. All time estimates began with the initiation of radiation treatment. Observation of response and toxicity was performed and toxicity was classified according to CTCAE Version 4.

## Results

### Response to treatment

The mean follow-up time was 8 months (range, 0–57 months). Cerebral follow-up imaging was performed in 46 patients. Six patients achieved a complete remission (CR) and 11 patients a partial remission (PR), while 11 patients showed an intracerebral stable disease (SD), 1 patient a mixed response (MR) and 17 patients an intracerebral progressive disease (PD). Remission lead to a significantly improved OS (p < 0.001) with median 13.5 months compared to 4.4 months in patients without remission and 2.4 months in patients without information on imaging outcome. Furthermore, the majority of patients were additionally treated with steroids for clinically symptomatic brain edema or significant brain edema seen in cerebral imaging procedures. Thirty-two patients reported improved symptoms. Twenty-four patients reported to be asymptomatic at end of treatment, 19 patients to have stable symptoms and 8 patients described worsening of symptoms.

### Survival

**Figure 1:**
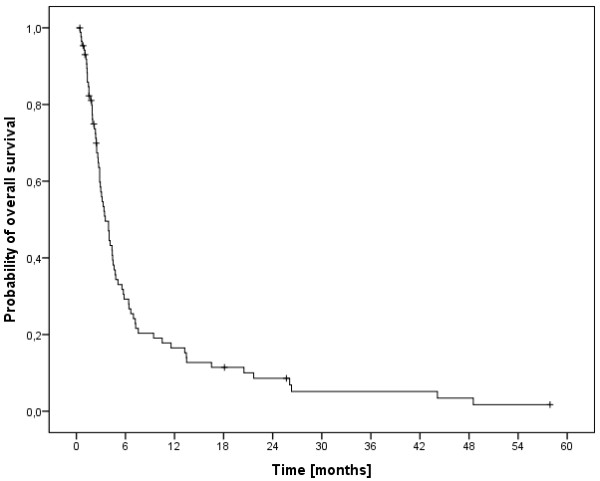
Kaplan-Meier estimation of overall survival (n=87).

**Figure 2:**
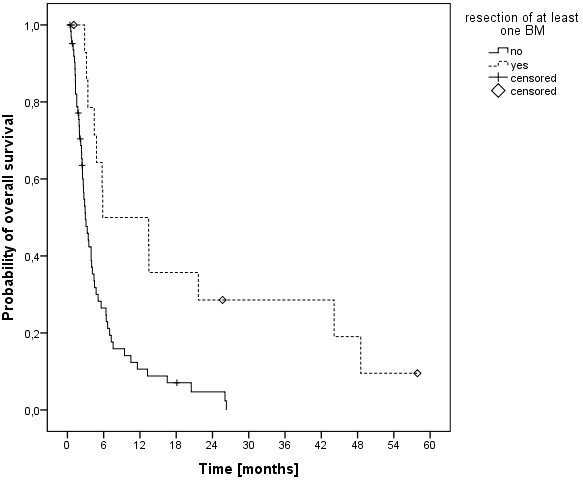
Kaplan-Meier estimation of overall survival in patients with (n=18) and without (n=66) prior resection of at least one brain metastasis.

**Figure 3:**
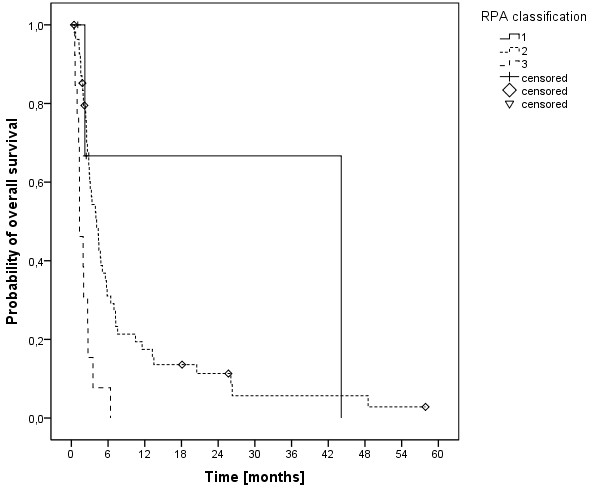
Kaplan-Meier estimation of overall survival according to the RPA classes (class 1, n=4; class 2, n=55; class 3, n=13).

**Figure 4:**
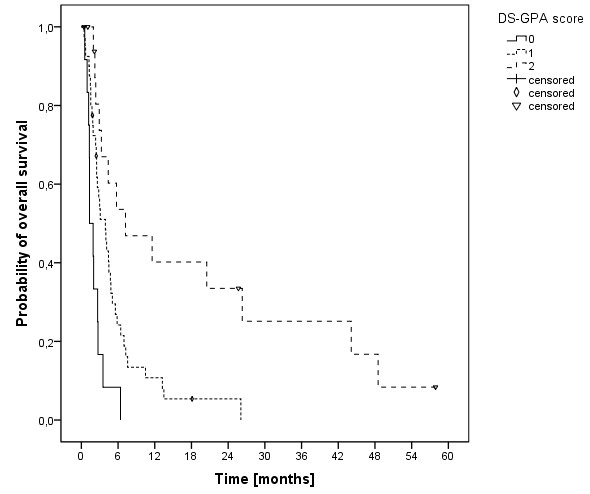
Kaplan-Meier estimation of overall survival according to the DS-GPA score (score 0, n=12; score 1, n=40; score 3, n=18).

**Figure 5:**
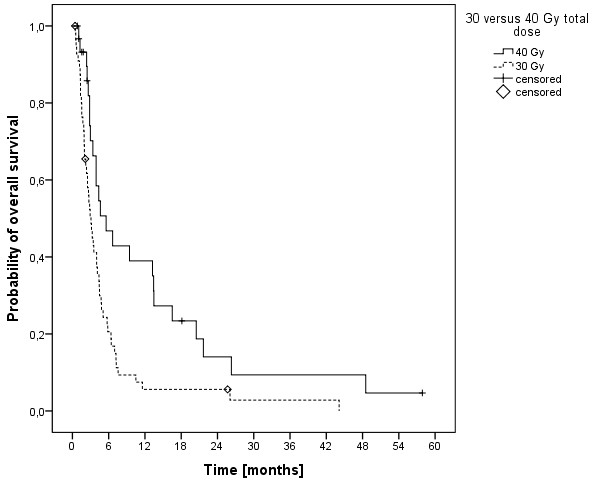
Kaplan-Meier estimation of overall survival in patients treated with 40 Gy in 20 fractions (n=31) versus 30 Gy in 10 fractions (n=56).

The median overall survival time was 3.5 months (range, 0.5-58 months) and the 6-, 12- and 24-months overall survival rates were 29.2%, 16.5% und 8.6%, respectively (Figure 1). At last follow-up, 9 patients were alive, while 78 had passed. The cause of death was documented as progressive disease in 11 patients (14.1%), intracerebral hemorrhage in 3 patients (3.8%), pulmonary embolism in 2 patients (2.6%), meningeosis melanomatosa in 2 patients (2.6%), respiratory failure in 2 patients (2.6%) and liver failure in 1 patient (1.3%). In 57 patients (73%) the cause of death was not documented. In univariate analysis, no significant influence on OS was seen for age (p = 0.314), gender (p = 0.729), Clark level (CL, p = 0.786, median CL III, analyzed CL >/= or < III), tumor thickness (TT, p = 0.646, 2.25 mm, analyzed TT >/= or < 2.25 mm) and having extra-cerebral metastases (p = 0.206, median 5.1 months without versus 3.2 months with). A significant influence on survival was seen in patients having had a surgical resection of at least one cerebral metastasis, while in 2 of these patients a single brain metastasis was resected (p = 0.001, median 5.8 versus 3.0 months, Figure 2). Chi-square analysis showed that patients with higher DS-GPA scores were more likely to have surgical resection of BM (Pearson, p = 0.022). On the other hand, patients were not significantly different distributed between the two different dose concepts regarding surgical resection prior to irradiation (Fisher exact test, p = 0.084) and salvage SRS (Fisher exact test, p = 0.286). Patients in RPA class I had a median OS of 44 months, in class II of 4.1 months and in class III of 1.3 months (p < 0.001, Figure 3). Furthermore, DS-GPA-score was prognostic (median OS in patients with 0 points 1.3 months, 1 point 3.9 months and 2 points 7.2 months, p < 0.001, Figure 4). A higher total treatment dose resulted in better outcome (p = 0.003, median 5.6 months vs. 3.1 months, Figure 5). Chi-square test showed that patients with DS-GPA scores 0–3 were not statistically significantly distributed between treatment groups with total doses of 30 Gy or 40 Gy (Pearson, p > 0.05). In multivariate analysis, total treatment dose (p = 0.042), surgical resection (p = 0.007) and GPA-score (p = 0.04) were prognostic. The results of the uni- and multivariate analyses were summarized in Table 2 and 3.

**Table 2:** Results of the univariate analyses (Log-rank, Mantel-Cox)

**Factor analyzed**	**p-value**
Development of side effects	0.596
Gender	0.729
Age (>/= vs. <58 years)	0.314
Clark level (>/= vs. < CL III)	0.786
Tumor thickness (>/= vs. < TT 2.25 mm)	0.646
Extra-cerebral metastases	0.206
RPA class	<0.001
DS-GPA-score	<0.001
Surgical resection of BM	0.01
Total treatment dose (30 vs. 40 Gy)	0.003

**Table 3:** Results of the multivariate analysis (Cox-regression)

**Factor analyzed**	**p-value**
Surgical resection of BM	0.007
DS-GPA score	0.04
Total treatment dose (30 vs. 40 Gy)	0.042
Age	0.7
Extra-cerebral metastases	0.52

### Side effects

Acute side effects at the end of the irradiation were available in 84 patients (97%). At the end of radiation treatment, 68 patients denied acute side effects, while 10 patients reported up to moderate (grade II-III) fatigue, and one patient each mild (grade I) and intense (grade IV) nausea, deficits in concentration and walking, brain edema, solitary headache as well as headache and dizziness. Long-term side effects were reported by two patients as fatigue and forgetfulness, respectively. The development of acute side effects did not significantly influence OS (p = 0.596).

## Discussion

This retrospective analysis reports on the treatment results of 87 patients treated between 2000 and 2011 for multiple cerebral metastases in malignant melanoma. The treatment consisted of a WBRT with a total treatment dose of either 30 Gy or 40 Gy. This analysis evaluates the outcome and toxicity as well as possibly prognostic factors to help finding ways to improve prognosis, morbidity and mortality in patients with multiple brain metastases in malignant melanoma.

The short median overall survival with 3.5 months is consistent with reports by Rate et al. and Stevens et al., who published a median survival after the development of brain metastases between 14 weeks and 5 months[14,16]. In the cohort of Rate et al., a factor associated with improved survival was surgical resection of a solitary brain metastasis, which led to a median survival of 36 weeks. The Analysis of Stevens et al. revealed complete macroscopic resection of all cerebral disease, absence of extra cerebral metastases and a single brain metastasis to be associated with improved survival. Long-term survival of > 2 years in 10 patients was associated with solitary brain metastasis and surgical resection. Comparable results were reported by Fife et al. [7]. In 2010 Eigentler et al. concluded that number of brain metastases (solitary versus multiple) and pre-treatment lactase-dehydrogenate (LDH) levels were primarily impacting on overall survival [26]. Unfortunately the pre-treatment LDH levels in our cohort were retrospectively not accessible, and additionally all patients except for two had multiple intracerebral metastases, since patients with one to three brain metastases from malignant melanoma are treated with SRS in our department. So our cohort represents patients with at least one negative prognostic factor: multiple brain metastases from malignant melanoma. One other factor influencing survival was RPA classification, which especially showed reduced survival in patients with RPA class III, while class I had the best prognosis, but one should keep the retrospective character of this analysis in mind. In the publication by Broadbent et al. on 474 patients with brain metastases −198 patients with brain metastases due to MM- survival in RPA classes I-III were 7.7 months, 4.7 months and 2.1 months, respectively [10]. This compared well with data reported by Gaspar et al., were survival in RPA classes I-III were 7.1 months, 4.2 months and 2.3 months, respectively [25]. Comparable results were seen regarding the DS-GPA score. As previously described by Sperduto et al. [27], patients with higher DS-GPA scores had significantly improved survival in our cohort. Furthermore, our data showed an increased survival for treatment with higher total doses; likely caused by the relative radioresistant biology. In the literature, Broadbent et al. reported a dose response with a median survival of 4.7 months for 30 Gy in 10 fractions and 3.0 months for 20 Gy in 5 fractions[10]. Further on, 42 patients were treated with doses above 30 Gy, which seemed to further improve survival (median 12.9 months). The hypothesis that patients with brain metastases from malignant melanoma might benefit from a dose escalation beyond 30 Gy was posed by Rades et al. [28]. In relative radioresistant tumors an increase in total dose might improve tumor control and thereby long-term outcome. Radiosurgery (RS) has shown to be able to achieve 1-year local tumor controls as high as 81% in brain metastases from malignant melanoma. The combination of RS and WBRT might reduce local failures as reported by Brown et al. or Rades and Schild even though other reports (e.g. Liew et al.) did not show improved local control by adding WBRT to RS [29-32]. Or after resection of a solitary brain metastases, WBRT + boost to the metastastic region increases local control, but not survival [33]. Still, the next step might be to perform a primary WBRT with integrated boost to the brain metastases. A phase one trial of simultaneous in-field boost with helical tomotherapy for 1 to 3 brain metastases did not show dose-limiting central nervous system toxicity [34]. So this approach might play a role in treatment of brain metastases from malignant melanoma in the future. Additionally the combination of WBRT and systemic treatment such as Ipilimumab might be a potential treatment approach in malignant melanoma and will be evaluated in a prospective randomized trial (ELEKTRA).

## Conclusion

The treatment of multiple brain metastases from malignant melanoma with WBRT is well tolerated and intracerebral tumor remissions could be achieved in several cases. A dose response with an advantage for higher total treatment doses was seen. However, treatment outcome is non-satisfying, and further improvements in treatment of brain metastases from malignant melanoma are warranted.

## Competing interests

The authors declare that they have no competing interests

## Authors’ contributions

HH: analysis and interpretation of data, writing manuscript. OD: critically revision for important intellectual content, interpretation of data. DH: acquisition and analysis of data. SR: critically revision for important intellectual content, interpretation of data. JD: critically revision for important intellectual content, interpretation of data. FS: critically revision for important intellectual content, interpretation of data. SEC: substantial contributions to conception and design; critically revision for important intellectual content; final approval for publication. All authors have read and approved the final manuscript.

## Funding

This research received no specific grant from any funding agency in the public, commercial, or not-for-profit sectors.
